# Minimally invasive complete mesocolic excision for right colon cancer

**DOI:** 10.1002/ags3.12331

**Published:** 2020-04-07

**Authors:** Hyunmi Park, Tae‐Hoon Lee, Seon‐Hahn Kim

**Affiliations:** ^1^ Department of Surgery Korea University Anam Hospital Seoul South Korea

**Keywords:** colon malignancy, colorectal cancer, D3 lymphadenectomy, mesocolic excision, minimally invasive surgery

## Abstract

Complete mesocolic excision (CME) with central vascular ligation (CVL) follows the same principles as the total mesorectal excision (TME) in the rectum of following the embryological planes for right‐sided cancers. The number of lymph nodes yielded increased with a resultant improvement in the oncological outcomes and by reducing local recurrence rates. Hohenberger's radical CME and CVL and the East's modified CME with D3 lymphadenectomy, which traditionally followed the embryological plane dissection for most of its intraabdominal cancer resection, have both shown to harvest significantly higher number of lymph nodes leading to a higher overall survival rate than the traditional right hemicolectomies of the West. To achieve the oncologically superior excision of the CME, awareness of the significant vascular anatomical variation will enhance the precision of the oncosurgery as well as minimize the risk of vascular complications. There has been an increasing body of evidence emerging on the safety of minimally invasive surgery (MIS); both its oncological safety as well as complication rates in the hands of expert and trained surgeons. The surgical technique of a CME right hemicolectomy is described step by step to aid standardization. There is mounting evidence that CME + CVL/ D3 improves survival in patients with colon cancer. Whilst the technical aspect of MIS is more challenging than the left, with a standardized technique and systematic teaching method, safety and benefits for patients can be achieved.

## Graphical abstract

This manuscript reviews the evidence of minimal‐access surgery in the treatment of right‐sided colonic cancers and describes a standardized technique for an Eastern modified complete mesocolic excision with D3 lymphadenectomy for the treatment of the right‐sided colorectal cancers.

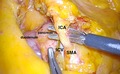

## 1 INTRODUCTION

The burden of colorectal cancer is increasing worldwide, and is currently the third most commonly diagnosed cancer and the fourth cause of cancer‐related deaths.^1^ The incidence of colorectal cancer is considered a marker of cancer transition, with rapid societal and economic advances resulting in its increase. The doubling of colorectal cancer in East Asia, such as in South Korea,^2^ Japan,^3^ and China, over the last two decades mirrors the economic growth in these countries. 

Right‐sided colon cancers are described as cancer of the cecum, the ascending colon up to the hepatic flexure and the proximal part of the transverse colon. Embryologically, the right side of the colon arises from the midgut, whilst the left arises from the hindgut. The transverse colon is composed of both structures, although more from the midgut rather than the hindgut. Over the last decade, research and publications are pointing out the differences between cancers arising from the midgut and hindgut.^4^ The right colon, arising from the midgut, tends to have more flat polyps than the left, which harbors the typical garden‐type polyp. Right‐sided tumors are also more likely to develop in patients with a genetic predisposition, such as those within the Lynch syndrome or microsatellite instability mutation.^5^ The differing responses to chemotherapy have been published in various studies,^6^, ^7^ with the right side fairing worse than cancers arising from the left side of the colon.

Since the introduction of total mesorectal excision (TME) in the rectum after the landmark paper by Heald in 1987,^8^ not only has the excision been standardized worldwide and the lymph nodes yield increased, but there has been significant improvement in oncological outcomes mainly attributed to the reduction in local recurrence rates, which, in turn, has had an impact on overall survival rates. The technique of following the embryological planes has also been adapted to right‐sided cancers in the West; in a 2009 paper, Hohenberger^9^ coined the term complete mesocolic excision (CME) with central vascular ligation (CVL) for this technique. The significant results from the Erlangen team showed a reduction in the 5‐year local recurrence rates from 6.5% down to 3.6%, and an increase in cancer‐related 5‐year survival rates from 82.1% up to 89.1%. The mounting body of evidence supporting the improved oncological effects of CME cannot be ignored and in some countries, such as Germany, CME has been included in the guidelines for the treatment of colorectal cancer.^10^

The Aim of this study is to describe a standardized technique and systematic teaching method to safely undertake a CME + CVL/ D3 lymphadenectomy as mounting evidence suggests its superior benefit for patients with right‐sided colon cancers.

## 2 EAST VS WEST

The uptake of the CME + CVL method for right‐sided cancers has not been as popular in the West as TME has been for rectal cancers; this is perhaps due to its perceived higher risk of complications and technical challenges when resecting around such potentially variable vascular anatomy.^11^ Traditionally, in Eastern countries such as South Korea^12^ and Japan, the notion of resecting along the embryological planes whilst harvesting the lymph nodes down to its central arterial root has been a longstanding surgical method practiced by the majority of cancer surgeons.

This long‐established anatomical classification of the extent of lymph node resection has been used in other intraabdominal cancer surgery, such as gastric cancer resections,^13^ with the systematic harvesting of lymph nodes, which have been grouped based on their location. The D number expresses the extent of lymphadenectomy, where D3 describes the complete dissection of all three regional lymph node stations: the pericolic, the intermediate, and the main. The Japanese Society for Cancer of the Colon and Rectum (JSCCR) Guidelines 2005 for the treatment of colorectal cancer,^14^ which has been regularly updated, with the latest guidelines published most recently in June 2019,^3^ aims to provide an evidence‐based standard of treatment strategies to reduce inter‐institution variation for the benefit of both healthcare professionals and patients. The publication of such consensus guidelines has attributed to the widespread practice of D3 dissection for colorectal cancer patients with a corresponding improvement in cancer survival. The 2014 paper by Ishiguro et al^15^ investigated the changes in colorectal care in Japan before and after the publication of the guidelines on D3 lymphadenectomy and the use of adjuvant chemotherapy. The data from 46 304 patients from 96 institutions showed that, from 2001 to 2010, the number of patients receiving D3 lymphadenectomies increased from 58.1% to 75% and the inter‐institutional variation on treatment was reduced. The authors highlighted the important role that the introduction of such guidelines made in accelerating the spread of high surgical standards.

The surgical concepts of the West's CME and the East's mesenteric mobilization both start with surgical separation, by sharp dissection, of the visceral fascial layer from the parietal fascia. The completely mobilized mesocolon is delivered with its intact visceral layer covering both sides, and with safe exposure of the ligation of the feeding arteries performed at their origin. The difference between the East and West arises during the right colonic mobilization, where Hohenberger's team^2^ advocate for kocherization of the duodenum with the pancreatic head. By doing so, mobilization of the colonic mesenteric root takes place down to the root of the superior mesenteric artery. Hohenberger also describes that, for cancer of the hepatic flexure of the colon, the greater curvature of the stomach should be freed 10‐15 cm from the arcade opposite to the tumor site, as about 5% of positive lymph nodes can be found over the head of the pancreas, and 4% of positive lymph nodes can be found along the gastroepiploic arcade at the greater curvature of the stomach.^2^ Following Hohenberger's ‘arcade principle’, the greater omentum is removed; however, in the East, only the omentum directly involved with the cancer is removed, en bloc with the tumor. The case of not removing an otherwise intact omentum arises from the fact that the arterial supply of the greater omentum is from the right and left gastroepiploic arteries (also called gastro‐omental), which, in turn, arise from the gastroduodenal artery on the right and splenic artery on the left. Both these arteries ultimately derive from the celiac trunk and none of the above vessels and their corresponding draining lymph nodes are known to be associated with colon cancers.^16^ Not completely removing an otherwise uninvolved omentum is also down to the important immunological and neovascularization properties of the omentum, which is well described in the literature.^17^, ^18^

In the East, the resection of the tumor, its mesentery, and the draining lymph nodes can be described as modified compared to the procedure in the West. The main aim of the operation is the harvesting of D3 lymph nodes and, to a lesser extent, the radical resection of the mesocolon; therefore, neither the duodenum nor the pancreas is mobilized fully as long as adequate lymphadenectomy is achievable.^19^

During left colon cancer surgery, the colonic mesentery mobilization is similar in both the East and West. The entire mesocolon of the descending colon and sigmoid, and the splenic flexure (required for an adequate, tension‐free length for the distal anastomosis) is mobilized off the retroperitoneal plane. When the surgeon dissects through the correct plane, the prerenal fat, the ureter, and gonadal vessels are not disturbed and left intact, allowing for a bloodless dissection through the intact visceral layer.

Even before the term CME was widespread, a Lancet retrospective observational study of pathologically graded colon cancer resection specimens by the Leeds team^20^ published in 2008 pointed out how dissecting along the right plane could improve survival, especially in patients with stage III colon cancers, in the same way that the TME could in the rectum. The paper noted a 15% (95% CI) overall survival advantage at 5‐years post‐surgery of mesocolic plane surgery compared with surgery in the muscularis propria plane (HR 0.57 [0.38‐0.85], *P* = .006), especially in patients with stage III cancers. The plane of surgery and amount of mesocolon removed varied between the different sites, with better planes in left‐sided resections compared to right‐sided ones.

The follow‐up paper in 2010 by the Hohenberger team^21^ in Erlangen from which the original CME and CVL paper^2^ was written, compared 49 CME and CVL resection specimens for carcinoma of the colon with a series of 40 standard specimens. The CME + CVL surgery resulted in specimens with greater amounts of tissue such as the distance between the tumor and the high vascular tie was higher, the length of the large bowel as well as the ileum were longer, and the area of the mesentery was greater. The lymph node yield was significantly superior with a mean difference of 30 vs 18 (*P* < .001) lymph nodes harvested with each specimen. This provides further evidence that grading the plane of dissection in colon cancer may be a valid and reproducible method of specimen assessment.

In Beijing, a paper by Gao et al^22^ showed results from a three‐year period on the efficacy and safety of CME. This study showed a statistically greater number of total lymph nodes retrieved in the CME group (24 vs 20, *P* = .002) as well as a greater area of the mesentery (both in the right colon and sigmoid resections) and tumor to high tie distance (right colon: 129 vs 113 cm; sigmoid colon: 143 vs 121 cm) without a difference in complication rates between the CME and non‐CME groups.

There is a dual purpose to the radical harvesting of lymph nodes during a CME and D3 lymphadenectomy down to its arterial root: the process is both for staging and therapeutic intention. The number of lymph nodes analyzed for staging colon cancers has been reported to be an independent prognostic variable outcome in itself. The improvement in survival of patients undergoing CME has also been attributed by ‘stage migration’,^23^ where patients are moved to a higher cancer stage postoperatively, requiring adjuvant therapy that may have been unforeseen during the preoperative staging. The high yield of lymph nodes results in a more accurate staging of the disease. Skip lymph node metastasis has been reported to occur in up to 18% of patients,^24^ where lymph node metastases do not spread in a step‐wise fashion from the paracolic to the intermediate to apical nodes, but can be present in the apical node alone with no presence in the other sites.

Both the West's CME + CVL and the East's D3 lymphadenectomy yield much larger numbers of lymph nodes than standard colectomies, which has been shown to be an independent positive predictor of long‐term outcomes. Colon cancer survival has been associated with an increasing number of lymph nodes analyzed even after controlling for the number of lymph nodes involved. The secondary analysis on the Intergroup Trail INT‐0089 on high‐risk patients with stage II and III colon cancers published by Le Voyer et al^25^ showed that even when no nodes were involved, the overall survival and cause‐specific survival improved the more lymph nodes were analyzed. Chang et al^26^ states that the number of lymph nodes evaluated after surgical resection is not only positively associated with survival of stage II and III colon cancer patients undergoing curative resection, but such evaluation could be a measure of the quality of colon cancer care. The American Society of Clinical Oncology’s (ASCO) 2018 post stated that the 5‐year survival for right‐sided tumors for both stage II and III were worse than left‐sided cancers. The 5‐year survival for stage II was 66% on the right compared to 70% on the left, and for stage III, it was 56% for the right and 60% for the left. But when 22 or more lymph nodes were harvested, the survival rate of right‐sided cancers was improved by 20%.

## 3 OPERATIVE AND PERIOPERATIVE COMPLICATIONS

There is significant variation of both the arterial and venous anatomy around the superior mesenteric artery,^27^ making the CME and D3 lymphadenectomy approach an inherently more challenging technique with a potential for catastrophic bleeding and other significant comorbidities such as injury to the duodenum or pancreas when compared to left‐sided cancers where there is more uniformity of the anatomy of the inferior mesenteric artery. The superior mesenteric vein (SMV) and its tributaries, including the Trunk of Henle, are potentially located around the arterial tree, making the exposure and arterial ligation, and harvesting of lymph nodes, technically hazardous. Further discussion on the variation of the arterial and venous anatomy of the right colon is dealt with under the technical notes in this paper.

A paper by the Copenhagen Complete Mesocolic Excision Study (COMES) Group and the Danish Colorectal Cancer Group (DCCG)^28^ published their short‐term outcomes, which compared CME with CVL to conventional colonic cancer resection. The study did not show any statistically significant increase in the 30‐ and 90‐day postoperative mortality, but there was an increased risk of injury to the spleen as well as to the SMV. There were also increased systemic postoperative complications such as sepsis, which required more than 24 hours of vasopressors and could result in respiratory failure.

The use of 3D imaging to delineate the vascular anatomy of the right colon preoperatively can provide surgeons with a precise view of the anatomy, which in turn could reduce the risk of catastrophic bleeding for patients undergoing minimally invasive surgery (MIS).^29^, ^30^ The authors agree with the published literature, that the use of preoperative imaging such as 3D‐CT could aid planning of CME with D3 lymphadenectomy for right‐sided colonic cancers, especially for the novice or training surgeon; however, its routine use is not common place at the authors’ institution, and not seen as mandatory. With experience and a systematic approach to the root of the mesenteric vessels, any variation of vascular anatomy can be tackled safely even during MIS as long as a clear view of the operative field is maintained, which in turn allows for secure vessel control in the event of inadvertent bleeding.

## 4 LAPAROSCOPIC VS OPEN

The widespread adaptation of MIS from the turn of this century for the treatment of colorectal cancer was aided by various landmark papers^31^, ^32^, ^33^, ^34^, ^35^, ^36^ such as the COST^23^ (Comparison of Laparoscopic‐assisted and Open Colectomy for Colon Cancer) trial, the CLASSIC^22^ (Conventional vs Laparoscopic‐Assisted Surgery In Colorectal Cancer) trial which included rectal cancers, and the COLOR^24^ (Colon Cancer Laparoscopic or Open Resection) trial which proved that laparoscopic surgery was as oncologically safe as open surgery. The overall message obtained from the short‐ and long‐term data showed no difference in the cancer recurrence rates after surgery but with positive short‐term outcomes in the laparoscopic groups. The surgical technique described in the above papers was the ‘traditional’ method prior to the conception of the CME, where the number of lymph nodes harvested was fewer than the current practice in East Asia. The evidence for the oncological safety of laparoscopic CME and D3 lymphadenectomy for right colon cancers is also mounting.

A large randomized controlled trial by Kitano et al^37^ of 1057 patients compared laparoscopic to open surgery, specifically Japanese D3 resections, for stage II or III colon cancer. The results showed no difference in overall survival between the groups, acknowledging the use of MIS in the treatment of such patients.

Negoi et al^38^ published a systematic review and meta‐analysis in 2017 involving a total of 12 studies with 1619 patients in the laparoscopic CME arm and 1477 in the open CME group which reported that the laparoscopic approach was offering the same quality of resected specimen as its open counterpart. There was no statistical difference in the number of retrieved lymph nodes nor in the distance between the tumor to the arterial high tie. The laparoscopic mesocolic specimen had a bigger surface compared to the open specimen. Not only did the laparoscopic CME with CVL produce the same long‐term, oncologically safe resection, but it showed itself to be superior in all the perioperative parameters.

Hahn et al^39^ published long‐term oncological outcomes for the more technically and oncologically challenging laparoscopic transverse colon cancer resections, which showed an average lymph node yield of 35.8 with minor complications only and no surgery‐related deaths. After a mean follow up of 40.5 months, there were no local recurrence rates, the overall survival was reported at 84.6%, and the disease‐free survival was reported at 89.3%. The laparoscopic technique was shown to be an oncologically safe approach when compared with previously published multicenter studies.

A large study by Shin et al^40^comparing 683 patients in the open modified CME and CVL group to 683 patients in the laparoscopic group used propensity score matching, adjusting for potential baseline confounders. The authors reported no significant differences between the groups with respect to age, gender, American Society of Anesthesiologist score, TNM stage, tumor size, lymphovascular invasion, and perineural invasion. There were no significant differences in postoperative morbidity (21.4% vs 18.3%, *P* = .175) nor mortality (0.1% vs 0%, *P* = 1.000) but the laparoscopic group had a shorter length of hospital stay. The 5‐year overall survival rate was 83.7% in the open group and 94.7% in the laparoscopic group (*P* < .001) with the laparoscopic group showing a significantly better 5‐year disease‐free survival rate (82.7% vs 88.7%, *P* = .009) and 5‐year disease‐specific survival rate (83.7% vs 94.7%, *P* < .001).

## 5 TECHNIQUE STANDARDISATION

In the 2018 review article by Hohenberger et al team, the need for teaching programs for minimally invasive CME to facilitate this technique was highlighted.^41^ Once the technical challenges have been overcome with thorough education, D3 lymphadenectomy for right‐colon carcinomas will become the standard of care, with the added benefits of MIS and its safe oncological outcome.

## 6 STANDARDISED MINIMALLY INVASIVE SURGICAL TECHNIQUE FOR RIGHT HEMICOLECTOMY (LAPAROSCOPIC/ ROBOTIC)

### 6.1 Positioning

The patient is laid supine on a tilt‐able operating table over a non‐slipping mechanism.

### 6.2 Incision

The open Hasson technique opens up the peritoneum under direct vision for the creation of a pneumoperitoneum commonly through the umbilicus. There is the option of creating the pneumoperitoneum with the use of a Veress needle insufflation if the surgeon is technically competent and the risk of adhesions is low as this blind access has the risk of bowel or organ damage. The rest of the ports are introduced under direct laparoscopic vision avoiding the inferior epigastric vessels and the bowel below. If the bowel resection and anastomosis is to be performed extra‐corporeally, only 5 mm ports are necessary; but if an endostapler is to be introduced, a 12 mm port is also required.

### 6.3 Exposure

After a general laparoscopy of the peritoneal cavity, the patient is positioned head down and right side up to take advantage of the gravitational force for better exposure. The omentum is moved over the liver, pushing the transverse colon away from the SMA root in the midline.

### 6.4 Vessel ligation

**FIGURE 1:**
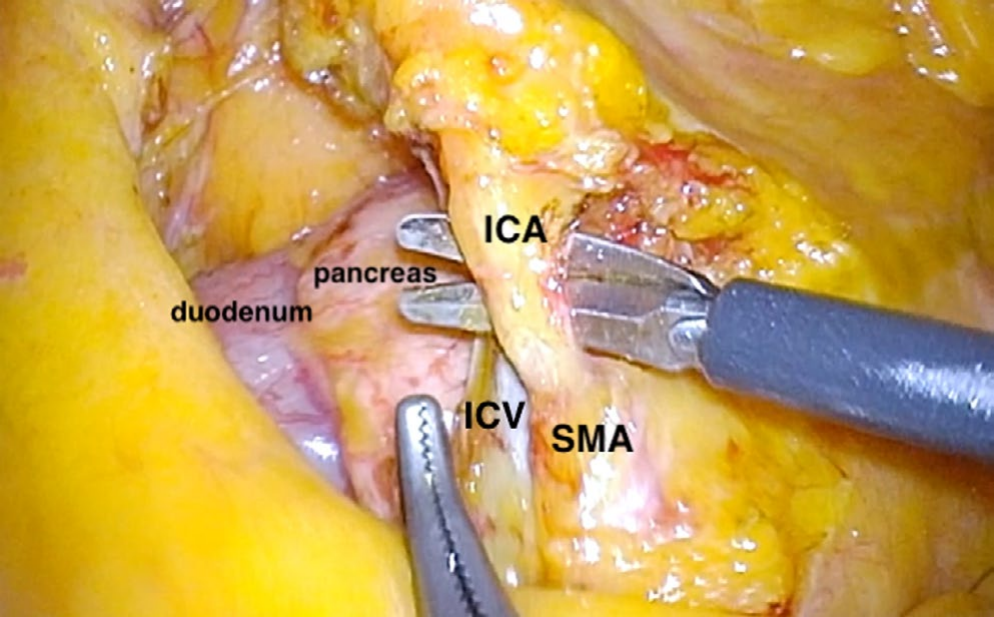
Isolation of ICA from SMA. ICA, ileocolic artery; ICV, ileocolic vein; SMA, superior mesenteric artery

**FIGURE 2:**
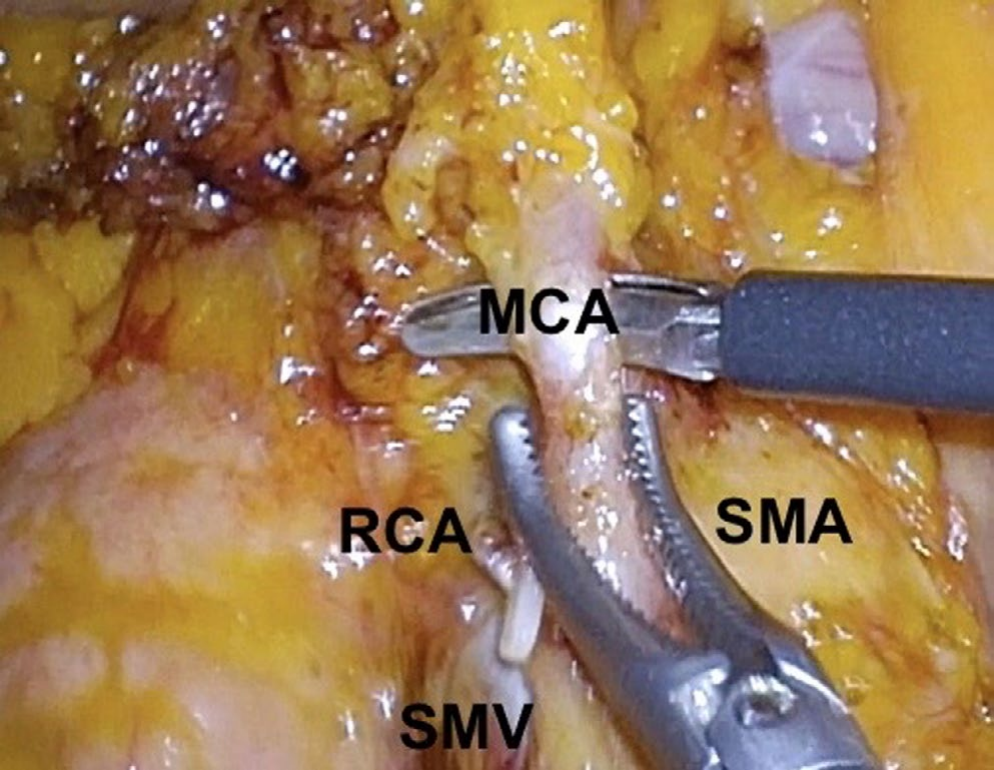
Isolation of MCA from SMA. MCA, middle colic artery; RCA, right colic artery; SMA, superior mesenteric artery; SMV, superior mesenteric vein

Identification of the ileocolic artery (ICA) root from the SMA in a thin patient is easier by looking at the grooves over the mesentery. In a more well‐endowed patient, lifting the ileocecal junction will reveal the ICA tenting posteriorly towards the SMA (Figure 1). Cephalad and anterior traction on the ICA (Figure 1), RCA and MCA (Figure 2) in the midline will reveal the SMA over which the mesentery is opened to reveal the base of each artery. This will allow for the successful harvesting of the lymph nodes around the vessels’ roots.

Unlike the traditional textbook description of the branches of the SMA being divided into the ileocolic, right (RCA), and middle colic arteries (MCA),^42^ there has been a constant stream of publications on the variability of the SMA irrigation of the right colon. In 1976, Vandamme and Van der Schuren^43^described from their 156 specimens that the RCA was only present in 13% of their abdominal preparations. This was reiterated in a publication by the Cleveland Colorectal Surgical Department^44^ in 1996, which combined data with reviews of published anatomic studies to conclude that, in the vast majority of cases, the SMA only has two independent branches supplying the colon. The right colic arising from the SMA was only present in 10.7% of the cases. A recent systematic review of cadaveric studies^30^ involving ten studies and 1073 cadavers, described how the anatomical variability of the RCA resulted in significant discord within the published literature. A systematic review and meta‐analysis of the surgical anatomy of the superior mesenteric vessels by Negoi et al^28^ showed that the venous drainage of the right side of the colon, which includes the infra‐pancreatic anatomy of the superior mesenteric veins, was also widely variable. The study found that the ICA and RCA had a trajectory posterior to the SMV in 57.4% and 10.6% of cases. This variation can contribute to the high risk of vein injury when the operating surgeon is trying to control bleeding from one of these pedicles, which can be retracted posteriorly to the SMV. The right colic vein (RCV) was present in only half of the cases and it drained equally to the SMV and trunk of Henle. The number of middle colic veins also varied as its drainage destinations. To achieve the oncologically superior excision of the CME for right‐sided colon cancers, being aware of this anatomical information will enhance the precision of the oncosurgery as well as minimizing the risk of vascular complications during such resection.

**FIGURE 3:**
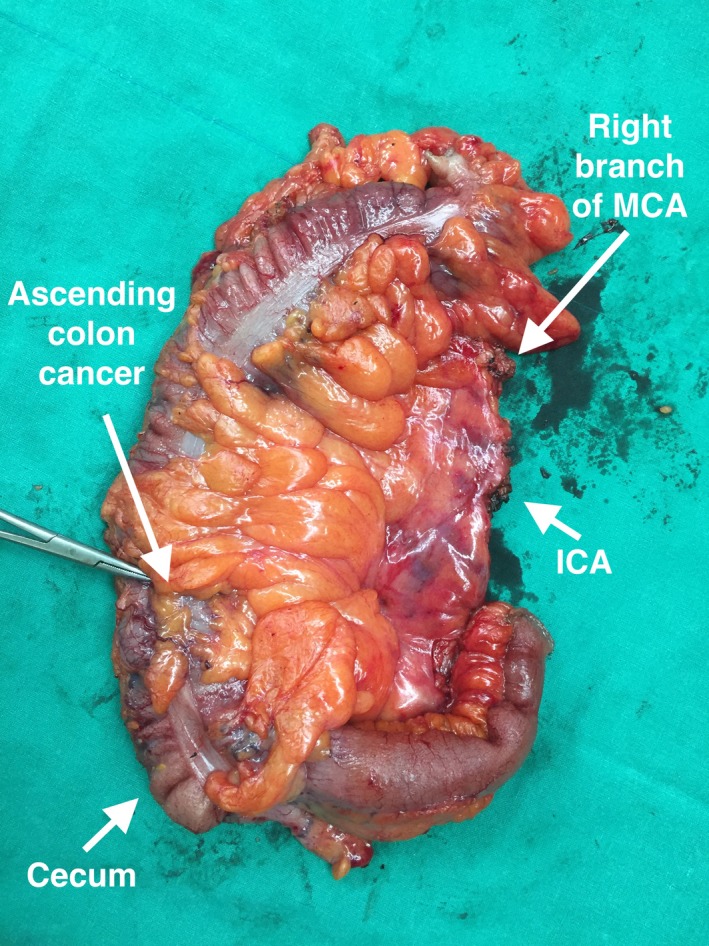
Ascending colon cancer. ICA, ileocolic artery; MCA, middle colic artery

**FIGURE 4:**
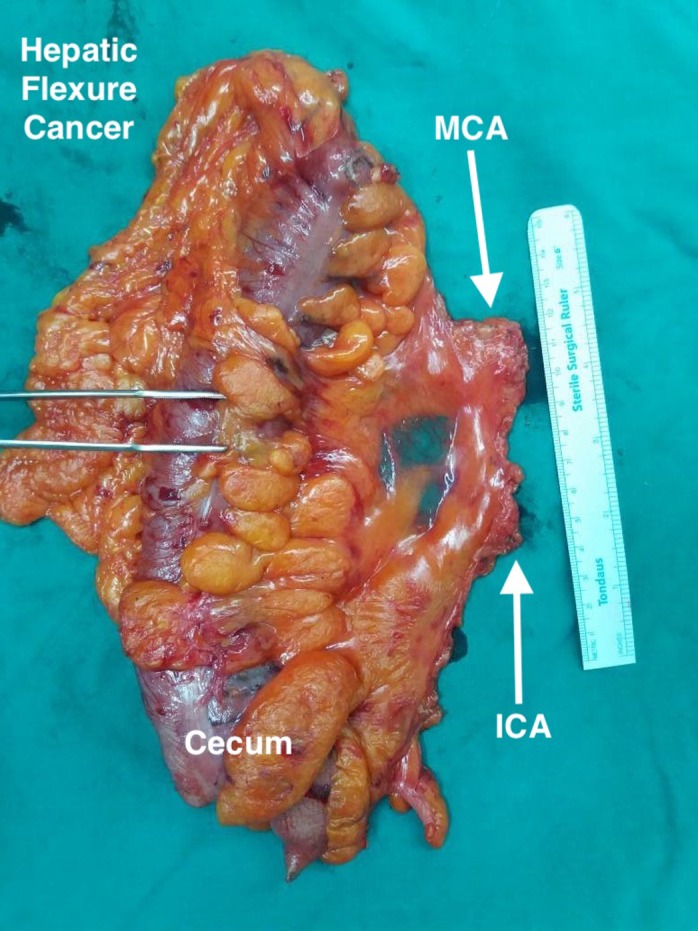
Hepatic flexure cancer front. ICA, ileocolic artery; MCA, middle colic artery

**FIGURE 5:**
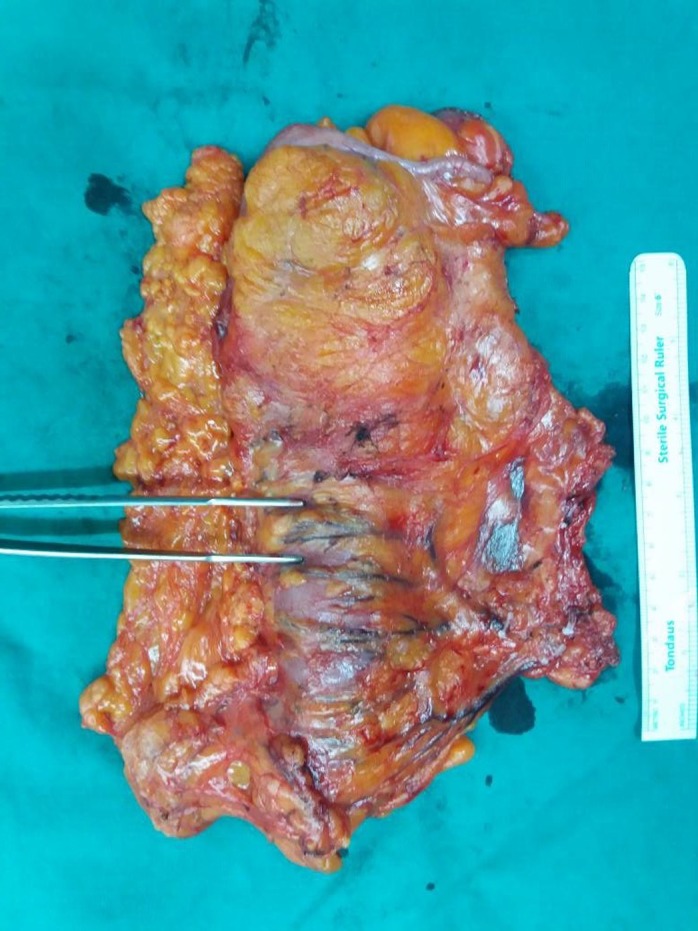
Hepatic flexure cancer back. ICA, ileocolic artery; MCA, middle colic artery

Figure 3 is an example of an ascending colon cancer, where only the right branch of the middle colic and its draining lymph nodes are needed to be harvested; however, in the case of more distal hepatic flexure cancers, such as the specimen in Figure 4 (front) and Figure 5 (back), the whole of the middle colic and its draining lymph nodes are harvested for the maximum oncological benefit that CME and D3 resection offers.

### 6.5 Medial to lateral dissection

Once the vessels have been divided with at least two endoclips on the patient's side, tunneling under the mesentery laterally takes place by dropping the duodenum and pancreas down and lifting the colon, separating the visceral and parietal fascia along the embryological planes.

Once sufficient medial to lateral dissection has taken place, the omentum is dissected off the proximal transverse colon towards the hepatic flexure.

The terminal ileum, caecum, and ascending colon are dissected off from the lateral peritoneal reflection to meet with the free edge of the hepatic flexure superiorly and the terminal ileum inferiorly. Caution must be taken during the medial dissection of the caecum and ascending colon as the right ureter has been known to be damaged when the mobilization plane is mistaken by a single layer.

### 6.6 Division of the colon

The length of bowel to be removed is dictated by the arterial supply of the right colon in parallel with the lymphatic drainage; therefore, enough length either side of the tumor needs to be dissected free and delivered out onto the wound. The current 2019 Japanese Society for Cancer of the Colon and Rectum (JSCCR) guidelines^3^ for the treatment of colorectal cancer recommends 10 cm as the optimal length of resection margin, as metastasis of the pericolic/perirectal lymph node at a distance of 10 cm or more from the tumor edge is rare. In the rest of the developed world, 5 cm lateral margins have been acceptable to reduce anastomotic recurrences.^45^

If the anastomosis is to be performed extra‐corporeally, the tumor is delivered to the skin through a mini laparotomy with the use of a wound protector with a ready‐made lid for re‐laparoscopy. Once delivered, the ICA vessel division site is identified, and the terminal ileum and transverse colon are divided with enough proximal and distal‐free margins. The corresponding mesentery is divided using either an energy device or traditional suture ties to secure hemostasis to the edges while not denuding the bowel edges from its mesenteric blood supply, as this will have an impact on the anastomotic healing.

### 6.7 Ileocolic anastomosis

The choice of a mechanical stapler or hand‐sewn anastomosis is as per surgeon preference as long as there is good supply at the bowel edges without tension. Due to the discrepancy of the diameter between the small and large bowels, a side‐to‐side anastomosis is favored over an end‐to‐end anastomosis. Care needs to be taken during bowel handling and its edges checked for bleeding when staplers are used before closure of the lumen. After a watertight anastomosis with a wide enough lumen is performed, the anastomosed bowel is returned to the peritoneum and the lid or glove put on the wound protector to restore the pneumoperitoneum for a re‐look laparoscopy.

An intracorporeal anastomosis may require less mobilization and a smaller extraction site wound but a higher level of laparoscopic technique. Both bowel ends are divided with the use of the endostapler to free the bowel containing the tumor. A small entrance is made in each arm of the terminal ileum and colon through which each arm of the endostapler is introduced, and a side‐to‐side ileocolic anastomosis is performed. The resulting defect requires endoscopic suture closure whilst minimizing spillage of gut content into the peritoneal cavity. A two‐layer continuous full thickness suture is recommended starting at the bottom of the wound to minimize spilling of bowel contents into the peritoneum.

A Cochrane systematic review on transverse vs midline incisions for abdominal surgery,^46^ as well as a systematic review and meta‐analysis examining the impact of incision on outcomes after abdominal surgery,^47^ showed evidence to suggest that a transverse incision was superior to a vertical incision in the short‐term pulmonary function as well as in long‐term incisional hernia rates. During an intracorporeal anastomosis, after the specimen is freed, the extraction site can be placed on the most cosmetically suitable site, such as a Pfannenstiel or either iliac fossa transverse incisions.

Once the specimen has been delivered through a wound protector, the lid is put back on to restore the pneumoperitoneum. A final laparoscopy is performed to suction any free fluid and check for hemostasis as well as any malrotation of the returned anastomosed bowel.

### 6.8 Closure

The sheath of the extraction mini‐laparotomy site and the 12 mm port site are closed with strong dissolvable sutures. The wound through which the bowel was delivered is washed with saline and the skin closed with either interrupted or subcuticular dissolvable undyed sutures. A long‐acting local anesthetic to all wounds will improve the pain score when the patient wakes up from the general anesthesia.

## 7 CONCLUSION

Both in the East and West the evidence is mounting for the radical dissection of the mesocolon along its embryological planes together with widespread lymphadenectomy to improve survival of colon cancer patients. The minimally invasive approach is not only proving to be a safe and feasible approach, but one with better short‐term recovery profiles and as oncologically beneficial as the open approach for right‐sided colon cancer.

The technical aspect of the minimally invasive approach is more challenging than the left and is exposed to operator variability; however, with a standardized technique and systematic teaching method, the safety and benefits for the patient can be achieved as successfully as in the open surgical approach.

## DISCLOSURE

Conflict of Interest: The authors certify that they have no affiliations with or involvement in any organization or entity with any financial interest (such as honoraria; educational grants; participation in speakers’ bureaus; membership, employment, consultancies, stock ownership, or other equity interest; and expert testimony or patent‐licensing arrangements), or non‐financial interest (such as personal or professional relationships, affiliations, knowledge, or beliefs) in the subject matter or materials discussed in this manuscript.
